# Bucking the trend: Population resilience in a marginal environment

**DOI:** 10.1371/journal.pone.0266680

**Published:** 2022-04-27

**Authors:** Gill Plunkett, Graeme T. Swindles

**Affiliations:** 1 Archaeology & Palaeoecology: School of Natural and Built Environment, Queen’s University Belfast, Belfast, Northern Ireland, United Kingdom; 2 Geography: School of Natural and Built Environment, Queen’s University Belfast, Belfast, Northern Ireland, United Kingdom; 3 Ottawa‐Carleton Geoscience Centre and Department of Earth Sciences, Carleton University, Ottawa, Ontario, Canada; Centre National de la Recherche Scientifique, FRANCE

## Abstract

Evaluating the impact of environmental changes on past societies is frequently confounded by the difficulty of establishing cause-and-effect at relevant scales of analysis. Commonly, paleoenvironmental records lack the temporal and spatial resolution to link them with historic events, yet there remains a tendency to correlate climate change and cultural transformations on the basis of their seeming synchronicity. Here, we challenge perceptions of societal vulnerability to past environmental change using an integrated paleoenvironmental and land-use history of a remote upland site in the north of Ireland. We present a high-resolution, multi-proxy record that illustrates extended occupation of this marginal locality throughout the climate oscillations of the last millennium. Importantly, historically-dated volcanic ash markers enable us to pinpoint precisely in our record the timing of major national demographic crises such as the Black Death and the European, Irish and Great (Potato) Famines. We find no evidence that climate downturns or demographic collapses had an enduring impact on the use of the uplands: either the community escaped the effects of these events, or population levels recovered rapidly enough (within a generation) to leave no appreciable mark on the palaeoenvironmental record. Our findings serve to illustrate the spatial complexity of human activity that can enable communities to withstand or quickly bounce back from largescale calamities. In neglecting to consider such local-scale variability in social and economic organization, generalized models of societal collapse risk overplaying the vulnerability of populations to long- and short-term ecological stressors to the detriment of identifying the social constraints that influence a population’s response to change.

## Introduction

To what extent has past climate change determined the course of past societal development? Recent history provides ample evidence of significant human mortalities following extreme weather, for example the 1931 Yangtze floods [1], the 1970 Bhola cyclone [2], the 2003 European heat wave [3] and climate-related food crises such as the 1984–5 Ethiopian famine [4]. Such extreme events may be stochastic features of the climate system, but they can also be generated by short-lived climate perturbations (e.g., due to volcanic eruptions) or symptomatic of longer-term climate oscillations [5, 6]. Interannual climate change has also been proposed as a trigger for significant pandemics in the Common Era, such as the 541–550 CE Justinianic Plague and 1347–1351 CE Black Death [7–9]. The potential ramifications of climate shocks are epitomized by the aftermath of the 1815 eruption of Tambora, Indonesia, believed to be responsible for global cooling in 1816 that ultimately led to food shortages in Asia, Europe and North America, outbreaks of cholera and typhus amongst weakened populations, political unrest and largescale migration [10].

It remains to be seen if recent and historical climate-related environmental catastrophes will have any enduring legacy on the cultural record that will be discernible to archaeologists in centuries to come, but their demographic impacts have nonetheless influenced discourse concerning the susceptibility of populations to both long- and short-term environmental perturbations and the causes of past cultural change. For instance, prolonged and repeated climate downturns associated with the Little Ice Age have been considered transformative phenomena for European societies through their impact on agriculture [11, 12]. Some contend that rapid climate change played a role in wider social turmoil through its impact on economies, political systems and population dynamics [13–15], and centennial-long collapses in pre- and early agricultural societies have also been attributed to climate change [16–22]. Environmental events may even have played a role in shaping worldviews and ideologies [23–25], thereby having a profound impact on human behavior and the cultural record.

Critiques of neo-environmentally deterministic theories of societal transformations have been effectively rehearsed elsewhere, highlighting the complex interplay of social, economic and political factors that shape the responses of societies to ecological problems [26–34]. Frequently, societies already experiencing intrinsic turmoil are more susceptible to environmental stresses, being ill-prepared or lacking adequate leadership to respond effectively to crises [e.g., 30, 35, 36]. General models of collapse, when applied with broad brush to large regions without consideration of the cultural intricacies operating across a range of spatial scales, commonly fail to address the unequal geographies of vulnerability, resilience and adaptability which can exist even within small geo-political entities [cf. 33, 34, 37], or indeed macro-scale variability in climate and weather [38]. There is a risk too that the seeming coincidence of cultural transformations with climate events makes causality a tempting explanation, and it requires large datasets and highly refined chronological control to scrutinize temporal relationships [39, 40]. The *perceived* fragility of certain landscapes today may also sway interpretations towards concepts of heightened social vulnerability [41].

Here we illustrate an example of long-term population resilience in the face of numerous environmental adversities that underscores the capacity of communities to withstand such stresses even in an ecologically fragile, economically unproductive and geographically remote (thus, “marginal” in multiple respects) locale in northeast Ireland. Although but one locality on an island perched at the edge of the North Atlantic, our example highlights the ability for human populations to continue their ways of life in spite of a host of broader natural calamities. It speaks to a resilience of communities frequently overlooked in narratives of environmentally-driven past societal collapse.

### A focus on Ireland

The last millennium was arguably a taxing time for people living in Ireland when viewed retrospectively. Against a backdrop of long-term climate swings between the Medieval Climate Anomaly and the Little Ice Age through to the Modern (post-1850) period, Ireland suffered many major epidemics and famines, most notably the 1315–7 European Famine, 1348–9 Black Death, 1740–1 Irish Famine and 1845–52 Great (or Irish Potato) Famine [42–46]. Warfare and frequent skirmishes between rival kin groups and military incursions from Britain, sometimes resulting in large death tolls, also helped check population growth, as did emigration to the New World, but the population continued to grow until it reached its acme (>8 million) immediately before the Great Famine. In addition, demography was impacted by large-scale immigration from England and Scotland following the 1609 Plantation of Ulster, instigating in some regions the push of native populations into more remote and economically marginal areas [47]. Along with these socio-political pressures, extensive levels of poverty left many vulnerable to environmental stresses, particularly in those areas where monoculture was practiced [45, 48, 49]. Such is the telescopic version of the social and environmental crises endured by the population of Ireland since the Middle Ages. In reality, the tribulations of the peasant were vastly different to those experienced by town-dwellers or by the privileged classes, and each event impacted different regions, social classes, identity groups, genders and age groups to varying degrees.

Given the extent of environmental and demographic changes in Ireland over the last millennium, we reconstructed the paleoclimate and land-use history of an upland site in northeast Ireland to examine the impact of climate and national demographic catastrophes on human activity at a marginal locality. By virtue of the high rainfall and low summer temperatures that are typical of Irish uplands, these locations are generally considered environmentally fragile from a land-use perspective–susceptible to even low-magnitude climate perturbations–and their abandonment in prehistoric times has been regarded as symptomatic of a climatically-driven failure of the subsistence economy [50, 51]. In the north of Ireland, mean annual temperature decreases by ~0.5°C per 100 m in elevation, air and ground frost days increase significantly, and precipitation (rain and snow) is generally greater [52], all of which have a significant bearing on the potential for successful crop-growing and grassland productivity in upland areas. The cool and wet meteorological conditions have contributed to the formation of extensive peatlands (bog) that have limited the nature of land-use in these areas, leading to a modern perception that they are unproductive and suitable only for sheep-grazing and commercial forestry plantations. Accumulating over thousands of years, these peatlands are highly sensitive to climate changes [53] and preserve within them important biological and chemical archives of paleoclimate and local to regional paleoenvironmental change. Furthermore, Irish bogs contain a wealth of volcanic ash horizons (cryptotephras) that greatly improve the dating of peat sequences, particularly where historically-dated ash layers are preserved [54].

## Materials and methods

### Study site

**Fig 1:**
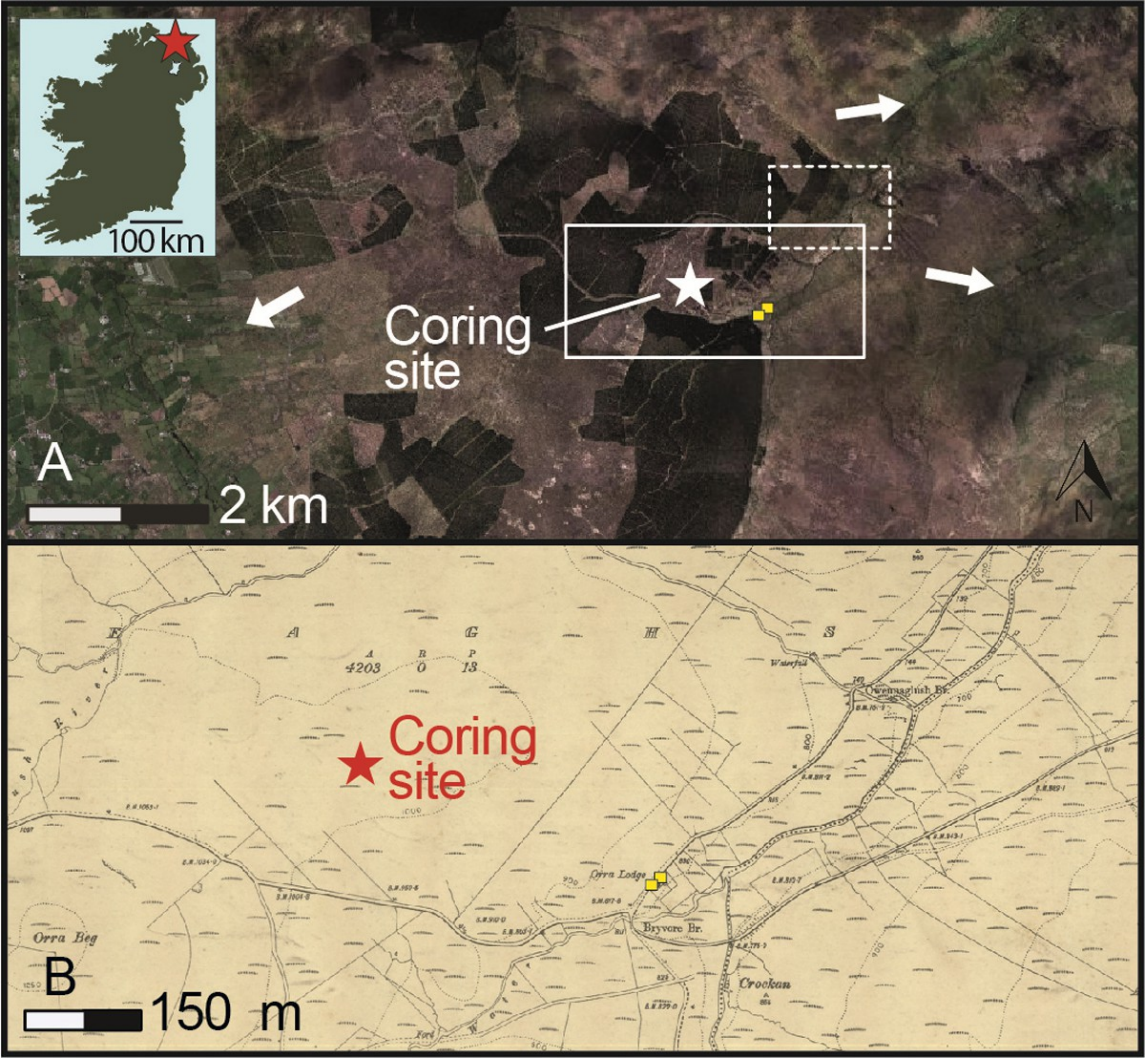
Location of Slieveanorra and the coring site. A) Aerial view of Slieveanorra (contains public sector information derived from Ordnance Survey Northern Ireland data and licensed under the Open Government Licence v3.0) showing the distance of the bog to farmland (indicated by arrows) today, and the position of tenant holdings (dashed box) and houses (points); B) Ordnance Survey map (2^nd^ edition c. 1906) showing historic settlement and land divisions near the margins of the bog.

Located at 300 m above sea level on the Antrim Plateau, Slieveanorra (55°05’04” N, 6°11’33” W) lies close to the present-day altitudinal limits for crop cultivation in the north of Ireland (Fig 1). This upland zone was specifically identified by Parry [55] as a climatically marginal environment vulnerable to crop failure during periods of climate deterioration. Our study site comprises a small raised bog, delimited to the north, west and south by elevated ridges now extensively covered in blanket peat or forestry plantations but which include abandoned habitations along a river valley to the east. Beyond this, the ground again rises, essentially limiting the main pollen source area to within about a kilometer of the sampling site.

For much of the last millennium, the landscape of the wider region was largely rural. The main town was Carrickfergus, 60 km to the south, established in the late 12th century following the Anglo-Norman conquest. In the low-lying hinterlands of the Antrim plateau, small towns and villages began to emerge only in the post-Plantation period, especially following the development of the linen industry in the 18th century. Today, the nearest settlements (mainly isolated farms and dwellings) are to be found on lower (<200 m above sea level) slopes along the river valleys 4–5 km to the north, east and west of the bog (Fig 1A). However, numerous abandoned houses, field systems and lazy-bed ridges (elevated strips of land separated by furrows to improve drainage for potato growing) are testimony to previous occupation of the river valleys in the immediate area of Slieveanorra Bog (up to at least 0.6 km from the bog; Fig 1B). The mid-19^th^ century Griffith’s Valuation indicates a concentration of six tenant holdings of just under 30 ha each along the river valley within 2.5 km to the northeast of the coring site, of which four are associated with farmers’ houses. Larger tracts of land (>750 ha), including that encompassing Slieveanorra Bog, were occupied by shepherds and cottiers (small farm tenants). At the time of the 1901 and 1911 censuses (earlier censuses for this locality do not survive), seven and six tenant households were occupied, respectively, the majority of which were engaged primarily in unspecified farming or shepherding [56]. The total population of the community was then in the order of 30–40 individuals, largely comprising the same families or their descendants who had been living there in the mid-19th century. By this time, the land was in the ownership of the Mulhollands, a family of linen manufacturers.

How early such settlement began, or whether occupation was always permanent, is difficult to gauge from the historical and archaeological records. The Down Survey of Ireland, a detailed land evaluation undertaken in the 1650s, classified the area as common (i.e., not privately-owned) and unprofitable land, describing it merely as “mossy bog” and providing no hint that it was occupied or cultivated. In the late 16th century, Slieveanorra was the site of a battle between the two rival Gaelic (native Irish) clans, which the defending clan won by luring the enemy onto treacherous bogland, but there are no references to local settlements. In the centuries before, the Antrim uplands had been surrounded by or encompassed within lands held by prominent Anglo-Norman families, following the conquest of the region in the late 12th century [57, 58]. Anglo-Norman land management in Ireland differed little from native practices except in its greater intensity, but was generally focused on lowlands (< 150 m above sea-level) [57].

### Field and laboratory methods

A peat core was collected at Slieveanorra bog in 2003 using a 5 cm-diameter Russian corer [59] as part of a multiproxy investigation of mid- to late Holocene paleohydrology [60–62]. A description of the bog is provided in S1 File. This study focuses on the uppermost part of the sequence spanning the last millennium, for which a highly resolved chronology is possible with the aid of historically dated cryptotephra horizons. Organic content of the peat profile was determined using the standard loss-on-ignition approach (combustion at 550°C for four hours [63]) on dried, contiguous 1 cm samples, enabling the calculation of inorganic (mineral) material blown onto the bog as a proxy for soil erosion in the catchment.

High-precision dating control was achieved using a combination of ^14^C dating, spheroidal carbonaceous particles (SCPs) and tephrochronology that together provided 17 dated horizons for age-modelling (see S1 Table in S1 File). The loss-on-ignition ash residues were treated with dilute hydrochloric acid, washed and mounted to identify the presence of cryptotephra horizons. Swindles et al. [62] have previously reported the identification of the historically dated Hekla 1947 and 1510 tephras at depths of 5–6 cm and 24–25 cm in the Slieveanorra core (data available at Tephrabase, www.tephrabase.org), as well as the decline of Spheroidal Carbonaceous Particles (SCPs) at 2–3 cm that comprises a marker horizon dating to AD 1980±3 [64, 65]. Additional cryptotephra horizons were re-subsampled and prepared for tephra geochemical characterization using the wet oxidization technique [66], and were then mounted in EpoxiCure epoxy resin on glass slides, which were ground and polished to expose the surfaces of the tephra shards. Major element geochemical analysis of glass component of the cryptotephras was performed on a Jeol FEGSEM 6500F at Queen’s University Belfast (QUB) or a Jeol 8800 superprobe at Oxford University (see S1 File for tephra identifications and S1 Dataset for analytical results, instrument settings, and secondary standard data). All geochemical data were normalized and compared to published and unpublished data in the QUB tephra database.

To supplement the chronology, samples of identifiable above-ground plant macrofossils and bulk peat were submitted for ^14^C dating to the ^14^Chrono Centre, Queen’s University Belfast, from selected depths. The combined dating information was used to construct an age-model for the profile using a P_sequence deposition model in OxCal 4.4 [67, 68] and the IntCal20 calibration dataset [69], incorporating a general outlier model for the ^14^C determinations. Further details of the age-modelling can be found in S1 File.

Samples for testate amoeba, plant macrofossil and humification analyses were taken at 1 cm resolution. The combined use of these methods from a single core circumvents issues of correlating the time of climate changes to human responses, at least in terms of land-use. Standard methods of preparation and analysis were employed [70–75]. Paleo-water-table depths were reconstructed from the testate amoeba data using the Northern Ireland [76] and the EU testate amoebae transfer functions [77]. Reconstructions were detrended by linear regression and expressed as residuals following Swindles et al. [78]. Humification data are presented as standardized light transmission values, lower values indicating a lower degree of decomposition [70]. Results were plotted in Tilia 2.0.41 [79, 80]. Raw data are included in S2 Dataset.

Samples for palynological analysis were extracted at 1 cm resolution. Tablets containing *Lycopodium clavatum* were added to each sample to enable pollen concentration calculations [81]. Samples were disaggregated using hot 10% potassium hydroxide, and were sieved through 120 μm and 6 μm polyester meshes to remove coarse and fine detritus respectively [82]. Samples were washed in alcohol before being transferred to vials containing silicon oil. Pollen was counted at ×600 magnification on an Olympus BX41 microscope, with critical identifications at ×1,500, and charcoal fragments, burnt *Sphagnum* remains and selected non-pollen palynomorphs (NPPs) were recorded simultaneously. Pollen identification was aided with reference to pollen keys [83, 84] and the pollen reference collection at the Palaeoecology Centre, Queen’s University Belfast. Pollen nomenclature follows Faegri & Iversen [83]. A minimum of 500 pollen grains was counted for each sample, including a minimum of 200 dryland pollen taxa (i.e., excluding bog taxa). Pollen accumulation rates (PAR; also known as pollen influx and expressed as grains cm^-2^ a^-1^) and charcoal influx were calculated using their concentration values (grains or fragments cm^-3^) and the peat accumulation rates determined by the profile’s age-model (S6 Fig in S1 File). Pollen percentages (S7 Fig in S1 File) were based on total dryland pollen. Pollen data are available from Neotoma (data.neotomadb.org/52205).

## Results

**Fig 2:**
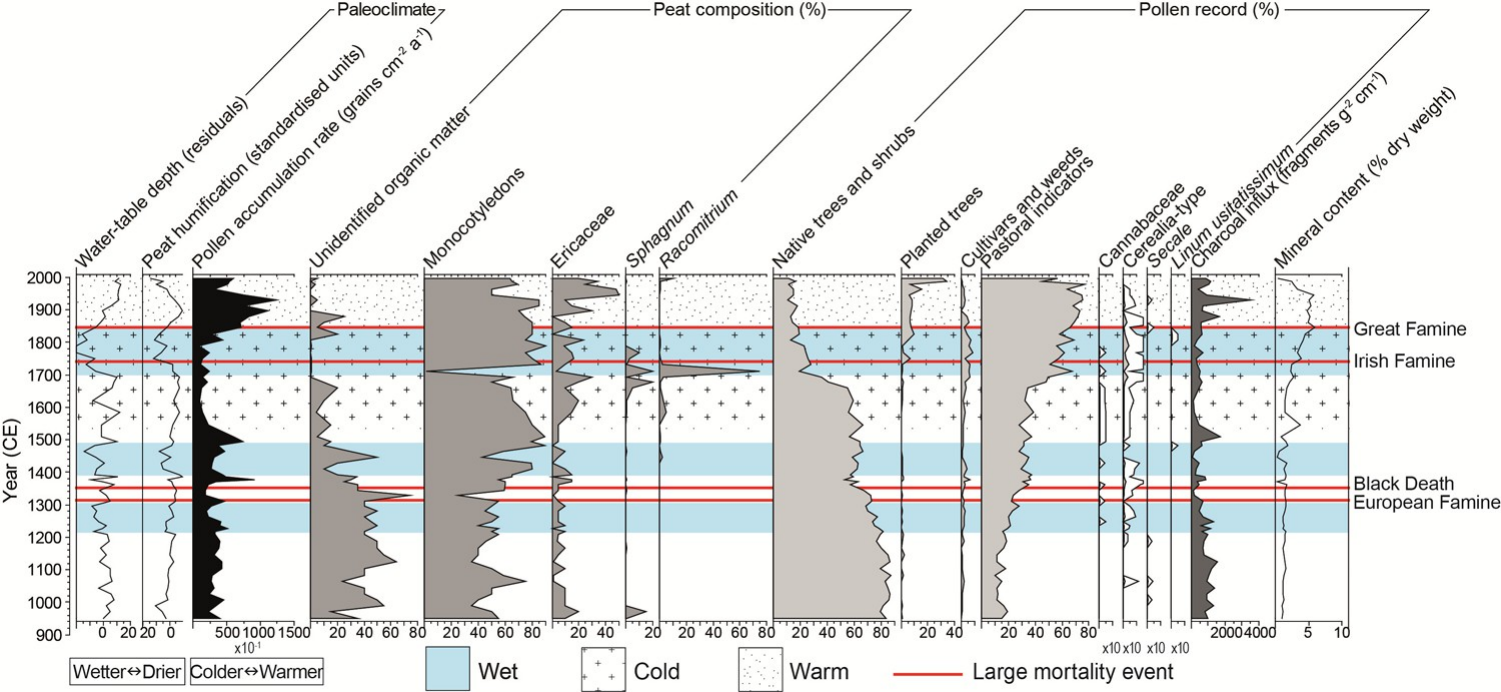
Summary diagram of paleoecological data from Slieveanorra. Phases of wetter bog surface conditions are highlighted by blue shading, and of changing temperatures by patterned stippling. Red lines denote the timing of major demographic crises in Ireland.

Fig 2 presents a summary of the key palaeoenvironmental results from Slieveanorra (see S1 File for full results). Paleoclimate proxies are represented by a testate-amoeba-derived water table reconstruction and peat humification, that signal changes in bog surface wetness, and PAR, that we use to infer changes in temperature. Bog surface wetness in ombrotrophic bogs such as Slieveanorra is a product of both precipitation and temperature; wetter conditions can therefore reflect colder and/or wetter climate predominantly in the spring/summer [85, 86]. As the water table reconstructions using the Northern Ireland and EU transfer functions indicate the same trends in water-table fluctuations (S4 Fig in S1 File), only the EU-derived curved is presented in Fig 2 for simplicity. High water levels will restrict the extent to which peat decomposes, which can be inferred from humification values. Pollen productivity has been correlated with summer temperatures of the previous growing season as well as flowering time (spring, summer) [87, 88]. Here, we infer changes in pollen productivity from the PAR. While total PAR may be driven by changes in vegetation with differential pollen production rates, changes in all main taxa and across all habitat groups signal a vegetation-wide response to an external factor. We observe in the Slieveanorra PAR two periods in which all dominant pollen taxa demonstrate coherent shifts (S6 Fig in S1 File), and interpret these as indications of changing summer temperatures. Local bog vegetation, represented by plant macrofossils, provides an additional window on bog surface conditions.

Human activity in the catchment is interpreted on the basis of arboreal (tree/shrub) to non-arboreal (excluding bog taxa) ratios, the latter representing more open conditions when tree-cover is reduced in the surrounding area. A range of crop, crop weed and pastoral indicators yields insights into the nature of land-use. We infer from such cultural indicators local occupation within the immediate surroundings of the bog, and expect that abandonment of farmed land will be expressed immediately by their decline and, within a decade or two, by an expansion of woodland taxa. Planted trees include exotic (e.g., fir, spruce, lime, beech) or locally extinct (pine) taxa known to have been introduced to Ireland from the 17^th^ century. Charcoal fragments in pollen slides are shown (expressed as charcoal influx), though the source of charcoal (natural or anthropogenic fires) cannot always be determined. Mineral content is presented as a proxy for soil erosion rates in the surrounding catchment.

Historically dated cryptotephra horizons provide exceptionally tight constraints for correlating the paleoenvironmental record with documentary evidence during this timeframe, notably with the identification of the Öraefajökull 1362 and Hekla 1845 tephras offering precise markers for examining the periods around the Black Death and Great Famine, respectively (S2 Fig in S1 File). These event horizons significantly reduce the chronological uncertainty that frequently hinders the reconciliation of paleoenvironmental events with documentary records. The age-model suggests an average peat accumulation rate of 17.8 yr cm^-1^, ranging from 7.9 yr cm^-1^ in the 14th century to 37.5 yr cm^-1^ between 1550–1660.

### Paleoclimate

From the opening of the record around 950 up until ~1200, the paleoclimate proxies from Slieveanorra Bog show limited fluctuations. This part of the record falls within the age-range of the Medieval Climate Anomaly, a period of general warmer conditions in Europe [89] although paleoclimate records from the North Atlantic region, including Ireland, show that this was a time of variable climate conditions [90]. Subsequently, testate amoeba-based water-table reconstructions and peat humification values signal three phases of wetter conditions, namely at ~1220–1300, ~1390–1490 and ~1700–1850 (Fig 2). PAR remains largely unchanged (less than one standard deviation of average values) during the first of these intervals, implying that the observed increases in bog surface wetness were driven chiefly by higher precipitation rather than by depressed summer temperature. Individual or successive extremes within these timeframes, such as the series of dry summers from 1252–4 or the summer drought of 1263 mentioned in the Irish annalistic records of the time [91, 92], cannot be discerned because of the decadal scale of the analysis. Similarly, extreme winters are poorly represented by the proxy records that have a bias towards environmental conditions during the growing season. A shift to lower water tables around 1300 appears to coincide, however, with a period of fewer reported extremes [92]. The severe cold years of 1315–6 that contributed to the European Famine have no expression in the Slieveanorra proxy records.

Bog surface wetness evidently increased again following ~1390, again in the absence of evidence for changes in temperature in the PAR record, and increased further in the late 1400s. This phase partly spans the Spörer Minimum, a period of reduced solar activity between 1460 and 1550 typically characterized in Northern Hemisphere paleoenvironmental records by cooler temperatures [92]. It is not until after the Spörer Minimum, however, that a temperature response is evident at Slieveanorra. A clear and sustained decline in PAR from ~1550 signifies a significant shift to colder summers that persists until ~1810, but bog surface wetness remains relatively low until ~1700, indicating cold and dry conditions until this time. This cold spell is reflected globally in paleoclimate reconstructions [93]. A considerable, albeit short-lived, expansion in *Racomitrium* moss at ~1700 points to a rapid onset of wetter conditions on the bog, as the moss is favored by a such a change following a period of prolonged dryness [94, 95]. Cold and wet conditions follow in the period ~1700–1850, corresponding with the main pulse of the Little Ice Age observed in other Irish paleoclimate records [96]. Our records lack the temporal resolution to highlight exactly the extreme cold years of 1740–1 that contributed to the Irish Famine, but this event evidently took place during a period of generally colder and wetter conditions. From ~1850, a substantial increase in PAR reflects Modern warming, and the bog surface becomes drier.

### Vegetation history and land-use

The vegetation around Slieveanorra at ~950 was predominantly wooded, illustrated by arboreal pollen in excess of 80%. It remained so until ~1150, although there are intermittent signs of human activity in the form of cereal-growing. From the late 12th century and through the subsequent climate oscillations, there is evidence of almost continuous occupation of this upland area until the early 20th century, represented by a sustained record of cultivars (Fig 2). The presence of cereal- and cannabis-type pollen, and more sporadically of flax, signifies local crop-growing–pollen from these cultivars does not disperse over long distances [97]–from which we can infer settlement, at least on a seasonal basis. Considering the topography surrounding the bog (blanket peat-covered slopes rising to the west and north), the most likely area of settlement was about 0.5 to 1 km to the north, south or southeast of the sampling site along the river valleys. The impact of this long-term occupation can be seen in the gradual decline of woodland (predominantly mixed oak-hazel) in favor mainly of grasslands. The 14th century famine and epidemic appear to have had no lasting (i.e., multi-generational) adverse impact on the level of land-use in the surrounding area, which instead increases from this time. This is not to say that inhabitants were not directly affected by such events, but the pollen record does not point to abandonment or waning land-use as a result of declining populations in the area around Slieveanorra. Many pollen records in north-western Europe show evidence for forest regeneration that reflects reduced land pressure following the Black Death [e.g., 98–101]. In Iceland and Scandinavia, population declines following epidemics saw the more marginal uplands abandoned in favor of lowlands [102, 103]. Inhabitants of the Slieveanorra area evidently withstood much of the climate variability during this time, the only break in occupation occurring in the mid-15th century which we infer from a decline in cultural indicators and an increase in arboreal pollen. A lapse in activity spanning a generation or two, and coinciding with a wet period, enabled some woodland regeneration, but farming resumed by the turn of the century. Agriculture persisted through the colder conditions of the 16th and 17th centuries, with elevated mineral content in the peat profile indicating increasing soil erosion.

From the mid-17th century, woodland declined severely in tandem with an increase in pasture and cereal cultivation, and implies that more land was cleared and taken into production. This change coincides broadly with the Plantation of Ulster, which prompted deforestation on a major scale both for timber resources and the destruction of refuges for the dispossessed rebelling against English and Scottish occupation [104–106]. The effects of this event can be seen in lowland pollen diagrams elsewhere in the north of Ireland [e.g., 107–109]. The Antrim region was not subject to direct plantation, and Down Survey records indicate that the uplands around Slieveanorra remained common land in the mid-17^th^ century. Land clearance at Slieveanorra at this time would therefore seem to have been instigated by the local population, whether in response to a greater demand for wood or because the population had grown. The shift to wetter conditions in the early 1700s coincides with a further reduction in woodland, but has no palpable impact on the extent of agriculture in the surrounding area. Brief and minor reductions in land-use occur around the time of the 1740–1 and 1845–52 famines, but neither calamity instigated a complete abandonment of the area. The extent of farming declined in the late 19th century, and finally ceased in the early 20th century. Subsequently, the abandonment of land, coupled with the establishment of commercial forestry in the immediate area, curtailed soil erosion rates, as inferred from decreasing mineral content.

## Discussion

The Slieveanorra pollen record demonstrates a long tradition of land-use in the Antrim uplands that can be traced back to the 12th century. Long-term occupation begins around the time of the Anglo-Norman conquest, but we have insufficient historical evidence to evaluate whether the activity represented in the pollen record was influenced, directly or indirectly, by Anglo-Norman activity in the region. A similar expansion of activity, including cultivation, is seen in pollen diagrams from elsewhere in the Antrim uplands at this time [110], which McNeill [57] regards as evidence of transhumance, a practice documented in Ireland from at least the first millennium CE [111]. Prior to the late 17th century, the scale of activity represented in the record is broadly comparable to that observed around the turn of the 20th century. Accordingly, we can envisage a level of agricultural land-use capable of supporting several family units; whether occupation was on a year-round or seasonal basis, it is not possible to determine with any certainty. It is evident, however, that the initial settlement of these uplands preceded the growth of population and waves of migration in post-Medieval and later times.

Insofar as the initiation of long-term settlement does not appear to coincide with any notable climate shifts in the paleoenvironmental record and continues despite oscillations in environmental conditions, climate does not seem to have been a limiting factor, or indeed a stimulus, for upland expansion. Examples of upland settlement initiation or expansion between the 10th to 13th centuries are also found in southwest Britain, Denmark and southern Sweden [100, 101, 112–114]. While the more clement conditions of the Medieval Climate Anomaly may have facilitated upland expansion, it cannot be seen as a specific “pull” factor, at least in the case of Slieveanorra, given that no seeming climate amelioration is evident in the 12th century. A comparative pollen study of lowland activity surrounding the Antrim Plateau region would help further the enquiry into potential circumstances influencing upland expansion.

Equally, the paleoenvironmental record conveys long-term occupation through several major climate fluctuations, including the severest centuries of the Little Ice Age, with the exception of the wet shift of the later 15th century. Sample resolution (mainly decadal to bidecadal) prohibits investigation of short-lived environmental or societal crises on individuals and is therefore too crude to detect the immediate impact of widespread pathogens and famines on the local population. The legacy of such events at a generational scale can be gleaned, however, enabling us to examine the population’s ability to absorb and recover from short-term catastrophes in the *longue durée*. Elsewhere in Europe, the significant mortalities and ensuing rural abandonment following the earlier 14^th^ century population crashes, aggravated by the Little Ice Age climate deterioration, are thought responsible for issuing in a late Medieval economic decline, a cessation of building activities and widespread woodland regeneration, which persisted into the 15^th^ century and thereby long-outlasted the events themselves [103, 113, 115–117]. Our highly refined chronology enables us to differentiate categorically the late 15^th^ century decline in activity at Slieveanorra from any early 14^th^ century events, and to track the extent of land-use throughout the climate oscillations of the Little Ice Age. Our findings add to an emerging body of literature that highlights that some “marginal” communities persisted through these times [118–121]. Similar resilience has been posited for the continuation of upland farming in Scotland through the Little Ice Age where agricultural productivity may simply not have been of prime importance to the occupants [41]. In Sweden and Norway, archaeological evidence suggests that community resilience was supported by versatile mixed economies amongst populations who were at a remove from feudal land organization and free to adapt to changing circumstances [119]. In Finland, historical records demonstrate that populations adopted innovative farming practices to mitigate the impact of climate on crop yields, and the farmsteads of tenant farmers were more likely to be deserted than those of peasant free-holders [122].

Faced with climate variability and extreme weather events, the longevity of occupation at Slieveanorra was almost certainly enabled by a mixed agricultural subsistence economy, perhaps supplemented by income from the production of hemp and linen, as well as freely available resources in the surrounding bogs, woodlands and rivers. We lack the archaeological record to investigate evidence for adaptability, but we assume that the inhabitants of the area successfully adjusted to the climate swings evident in the Slieveanorra paleoclimate record as the area continued to be occupied. More generally, a mixed economy combining subsistence farming with cottage textile industry helped buffer northeastern Ireland from the devastation of the potato blight and the resultant Great Famine that afflicted other regions [123]. The comparative geographic isolation–which might be regarded today as socio-economic marginality–of the community may have served to protect it somewhat from the pandemics that decimated inhabitants of urban and more densely-populated rural areas. In reality, the inhabitants are likely to have maintained familial ties with communities further down the valley, as suggested by the common family names recorded in the 19th and 20th century historical sources, and may not have perceived themselves to be isolated. If large-scale mortalities occurred, the population evidently recovered sufficiently from such tragedies not to leave a lasting trace in the paleoenvironmental record. It might be argued that the area became only truly became “marginalized” in the early 20th century, when the lack of modern infrastructure or social opportunities were likely greater “push” factors in upland abandonment than any environmental constraints, coupled with the “pull” of employment and other opportunities in growing towns such as Belfast. Thus, the factors that drove people to occupy this remote landscape, and to abandon it, should be sought primarily in the social domain.

## Conclusions

Climate variability, extreme weather and environmental catastrophes have undoubtedly been significant factors in demographic crises in the past, both directly and indirectly. However, previous studies have emphasized that is predominantly social constraints–notably factors such as urbanization, stringent modes of resource governance, social inequality and monocultures–that render a population most vulnerable to ecological crises and thus impact on its long-term resilience [27, 48, 124, 125]. Our findings reveal that the community occupying this seemingly remote and marginal environment in northeast Ireland was resilient (*sensu* [28]) to a range of climatic, environmental and social challenges during the last millennium.

What does this example contribute to our wider understanding of past societal vulnerability to ecological stresses? Our generationally-resolved, integrated climate and land-use record provides unequivocal evidence that, while the Little Ice Age climate perturbations were manifest in what is today a remote and marginal landscape, occupation persisted. Although the pollen record cannot point to adaptive strategies employed by the occupants of our study area, we argue that our evidence of persistent occupation speaks to the overall success of the local population in overcoming the challenges associated with both long- and short-term environmental swings, thus showing their resilience in the face of environmental shocks. Whether subsistence strategies were modified in response to changing conditions, we cannot determine, but the communities evidently endured: the climate downturns did not simply force abandonment of the uplands. Like studies in other regions of north and northwestern Europe, our case study illustrates the capacity of populations to withstand major environmental changes that, we propose, is enabled by a broad economic base that facilitates adaptability.

Our study highlights the inadequacies of macro-scale models in recognizing local-scale complexities in socio-economic structures and their susceptibility to ecological events. However widespread an environmental phenomenon (e.g., multi-regional in the case of events such as the Black Death or the Little Ice Age), not all communities are impacted in the same way, and if some communities persist, then population levels can recover and traditional ways of life can continue. Although the historical record clearly demonstrates large-scale calamities following extreme environmental events, their impact may well stem primarily from the rigid social structures that existed in many regions at this time; as such, they may be poor analogues for less hierarchical societies in which communities had greater capacity to adapt to circumstances. This has clear implications for understanding the transformative potential of past environmental crises, and in particular for explaining multi-centennial cultural “lulls” and “dark ages” and their relationship to demographic collapses. Without due consideration of the social conditions that rendered populations vulnerable to environmental events, we risk overstating the impact of environmental stressors and failing to identify the true causes of past cultural collapse that might help us to address today’s societal challenges in the face of environmental change (cf. [30]).

## Supporting information

S1 File Supplementary Information on the study site, chronology and age-model, and full suites of proxy data. (DOCX)

S1 Dataset Major element geochemical datasets for previously unpublished tephras from Slieveanorra. (XLSX)

S2 Dataset Plant macrofossil and testate amoeba raw data. (XLSX)
