# Supplementary material for: Bucking the trend: Population resilience in a marginal environment
Source: PLoS One. 2022 Apr 27;17(4):e0266680. doi: 10.1371/journal.pone.0266680 (PMC9045639; doi:10.1371/journal.pone.0266680)
Supplement: S1 File — (DOCX) [file pone.0266680.s001.docx]

Supplementary Information for

**Bucking the trend: Population resilience in a marginal environment**

Gill Plunkett & Graeme T. Swindles

Gill Plunkett

Email: [g.plunkett@qub.ac.uk](mailto:g.plunkett@qub.ac.uk)

**This file includes:**

Supplementary text

S1 to S7 Figs

S1 Table

References for SI citations

Supplementary Information Text

1.0 Study site

Slieveanorra (55°05’04” N, 6°11’33” W; 306 m above sea level) comprises an area of deep upland raised bog surrounded by a blanket bog complex in the Slieveanorra Nature Reserve, Co. Antrim. The Reserve is located on the Antrim Plateau, a massif topped by Palaeogene-age basalts characterized by steep glens that descend to the coast to the east. Rainfall generally exceeds 1,500 mm per year, and average temperatures range between 4–4.5˚C (January) to 14–14.5˚C (July). Mean annual potential evapotranspiration at this site is less than 350 mm.

The bog is set within a wider area of upland blanket bog that, since the 1950s, has been extensively planted with sitka spruce (*Picea sitchensis* (Bong.) Carrière). The surface of the bog features a complex of large pools containing aquatic *Sphagnum* species (notably *S. denticulatum* Brid. with *S. cuspidatum* Ehrh. ex Hoffmaround at the pool edges) and *Menyanthes trifoliata* L., surrounded mainly by very wet lawns characterized by *Sphagnum* species (mainly *S. capillifolium* (Ehrh.) Hedw. and *S. magellanicum* Brid.) and *Drosera* species.

S1 Fig illustrates the sheltered nature of the bog, surrounded by rising slopes to the north, south and west. Prevailing winds are from the west to south-west. The bog is delimited by the Owenaglush River to the north, and the Bryvore Water and Glendun Rivers to the south and south-east.


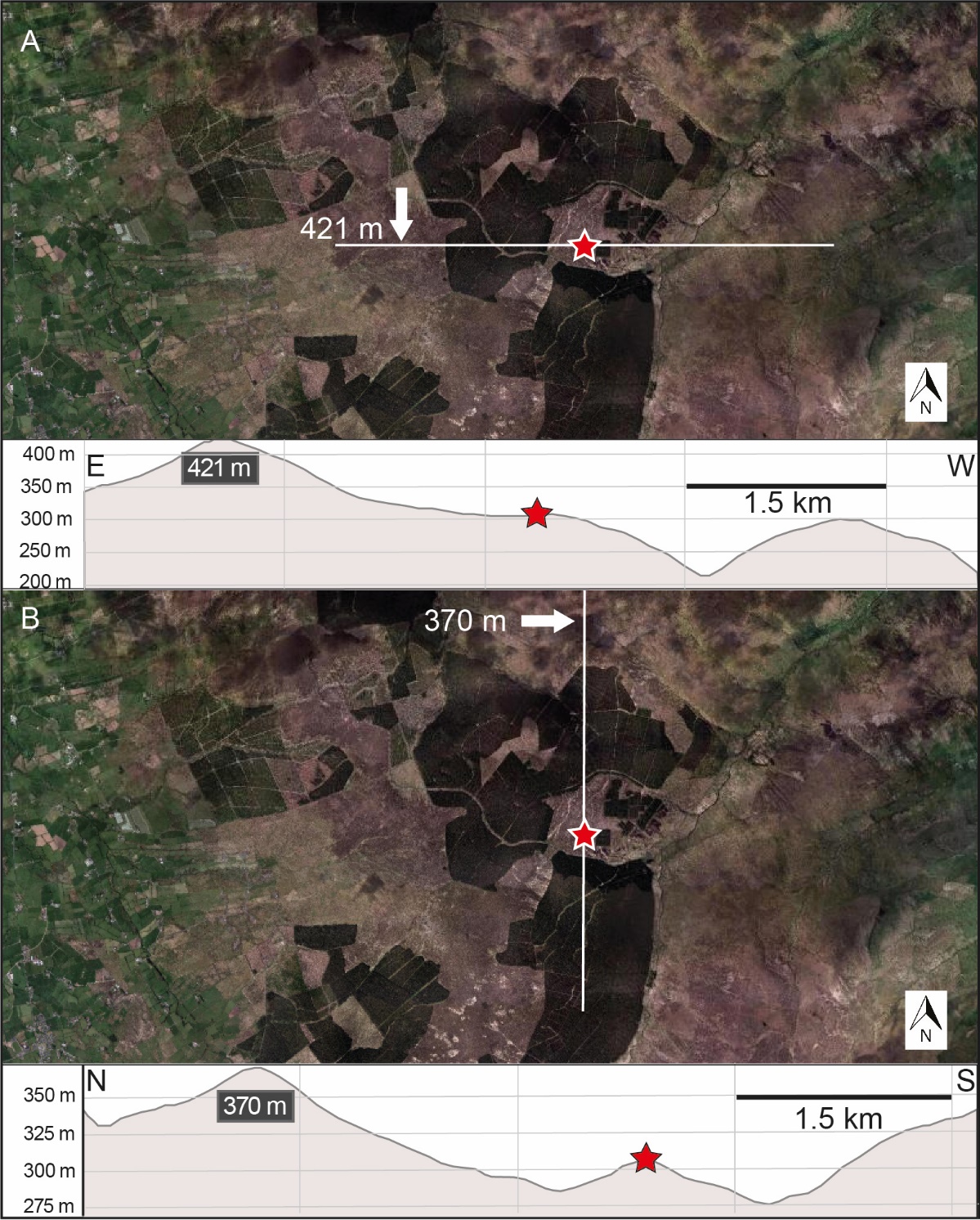


**S1 Fig**. Elevation profiles (based on Google Earth data) across Slieveanorra Bog and the neighboring area, showing A) east-west transect and B) north-south transect through the coring site (indicated by star). Slopes to the north, south and west are now under plantation forest (indicated by dark green areas) but were formerly covered by blanket pea (extant peatland represented by brown areas). Base maps contain public sector information derived from Ordnance Survey Northern Ireland data and licensed under the Open Government Licence v3.0.

2.0 Dating and age-modelling

Tephra shard counts for the Slieveanorra profile were converted to concentrations (shard g^-1^ dry weight; the tephrostratigraphy is presented in S2 Fig. Tephra peak concentrations at 4–5 cm and 24–25 cm were analyzed and reported by Swindles et al. [1]. A further 10 levels of peak shard concentration were sampled for geochemical analyses, five of which yielded sufficient shards for analysis (S1 Dataset). The geochemistry of the crytoptephras at 8–9 cm (QUB-585) and 10–11 cm (QUB-586) are consistent with the dacitic to basaltic glass end-members of Hekla. The lower layer is here interpreted to be the product of the Hekla 1845 eruption, in view of its co-occurrence with the start of an SCP curve dated to 1845±25 [1]. The upper layer likely represents upward vertical smearing of tephra shards. The peaks at 33–34 cm (QUB-522) and 35–36 cm (QUB-523) similarly produced inseparable geochemical suites identifiable as Öraefajökull 1362. The higher shard concentrations at 35–36 cm prompt us to consider this as the primary horizon. Finally, a small number of analyses (QUB-517) from a prominent peak in shard concentrations at 51–52 cm can be reliably attributed to Hekla 1104 found widely in the north of Ireland [2].

Samples for ^14^C dating were taken to supplement the chronology. Where possible, identifiable above-ground plant macrofossils were extracted for dating, but for some levels, the absence of such remains required that bulk peat samples be taken. At 20–21 cm (mid-point 20.5 cm), paired bulk and plant macrofossil samples yielded ^14^C ages that were indistinguishable, suggesting no significant offset in their ages (cf. [3, 4]). The results were incorporated with the tephra horizons and accepted date of 1980±3 for the decline of Spheroidal Carbonaceous Particles (SCPs), as identified by Swindles et al. [1], for Bayesian age-modelling using the P_Sequence in OxCal [5, 6], a general outlier model for the ^14^C determinations and the INTCAL20 calibration dataset [7], with the two dates for 20–21 cm combined within the model. A summary of the dating information is provided in S1 Table and the resulting age-model is presented in S3 Fig.


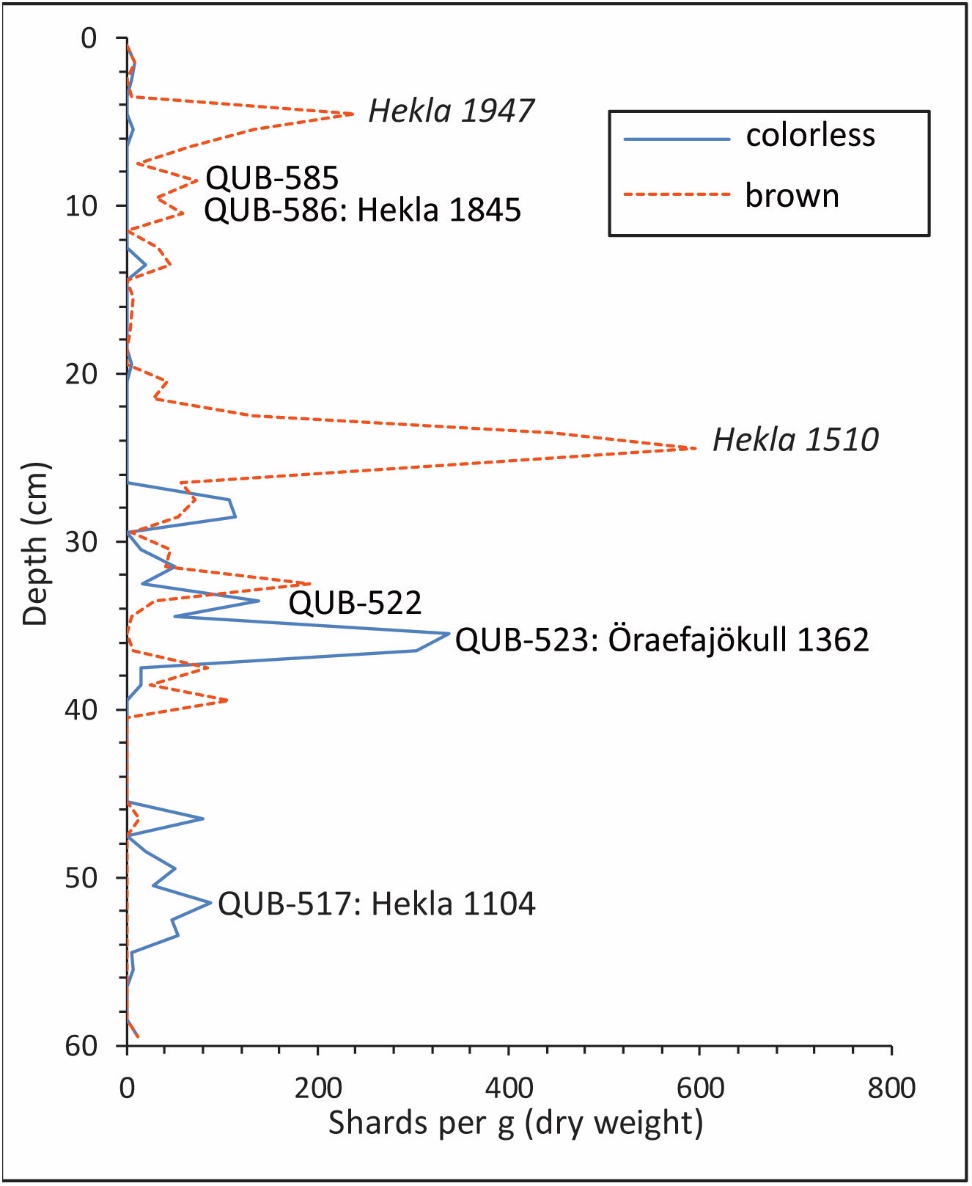


S2 Fig. Tephrostratigraphy for the upper 60 cm of the Slieveanorra core. Data are presented as number of shards (colorless or brown) per gram of dried peat. The position of the newly-identified cryptotephras (and lab identifier) used in the Slieveanorra age-model, along with the Hekla 1947 and 1510 cryptotephras identified by Swindles et al. [1], are shown.

**S1 Table**. Dating information used in Slieveanorra age-model. ^14^C samples were based on above-ground plant macrofossils unless otherwise indicated.

| Mid-depth (cm) | Dating method | Marker code | Calendar date CE | ^14^C date BP ± 1σ |
| --- | --- | --- | --- | --- |
| 0 | Date of coring | Surface | 2003 |  |
| 2.5 | SCP | SCP decline | 1980±3 |  |
| 4.5 | Tephra | Hekla 1947 | 1947 |  |
| 10.5 | Tephra | Hekla 1845 | 1845 |  |
| 16.5 | ^14^C (bulk) | UBA-7727 |  | 151±37 |
| 18.5 | ^14^C (bulk) | UBA-7728 |  | 177±22 |
| 20.5 | ^14^C (bulk)* | UBA-7729 |  | 195±26 |
| 20.5 | ^14^C* | UBA-17195 |  | 239±27 |
| 21.5 | ^14^C | UBA-16978 |  | 294±21 |
| 24.5 | Tephra | Hekla 1510 | 1510 |  |
| 26.5 | ^14^C (bulk) | UBA-7730 |  | 370±49 |
| 31.5 | ^14^C | UBA-32550 |  | 635±41 |
| 35.5 | Tephra | Öraefajökull 1362 | 1362 |  |
| 42.5 | ^14^C | UBA-32551 |  | 853±35 |
| 46.5 | ^14^C | UBA-17196 |  | 797±28 |
| 51.5 | Tephra | Hekla 1104 | 1104 |  |
| 52.5 | ^14^C (bulk) | Beta-208753 |  | 870±40 |
| 59.5 | ^14^C | UBA-32552 |  | 1137±32 |
| 99.5 | ^14^C (bulk) | Beta-207580 |  | 1680±40 |

*Dates combined in OxCal model


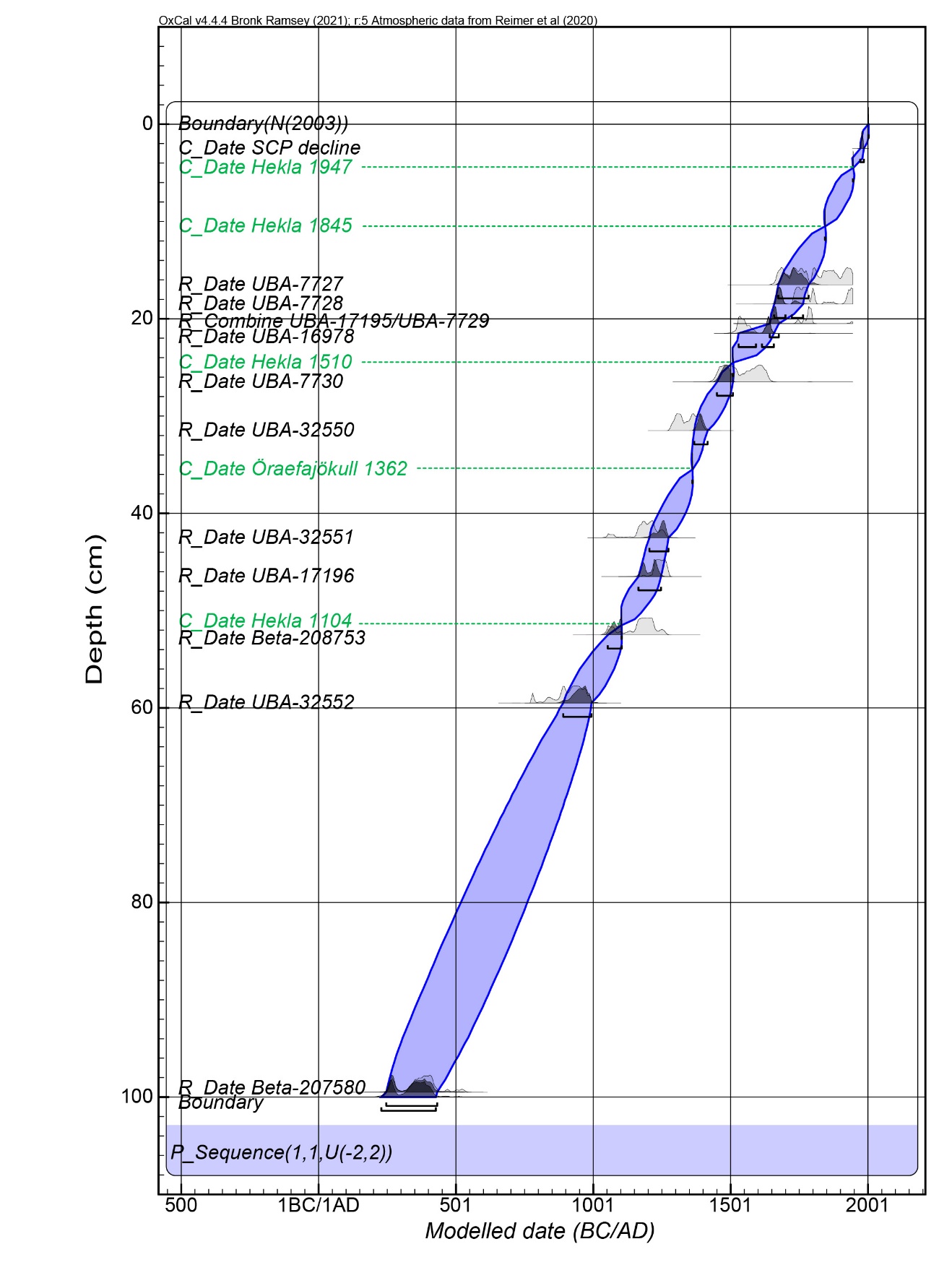


**S3 Fig**. **OxCal age-model for the upper 100 cm of the Slieveanorra core**.


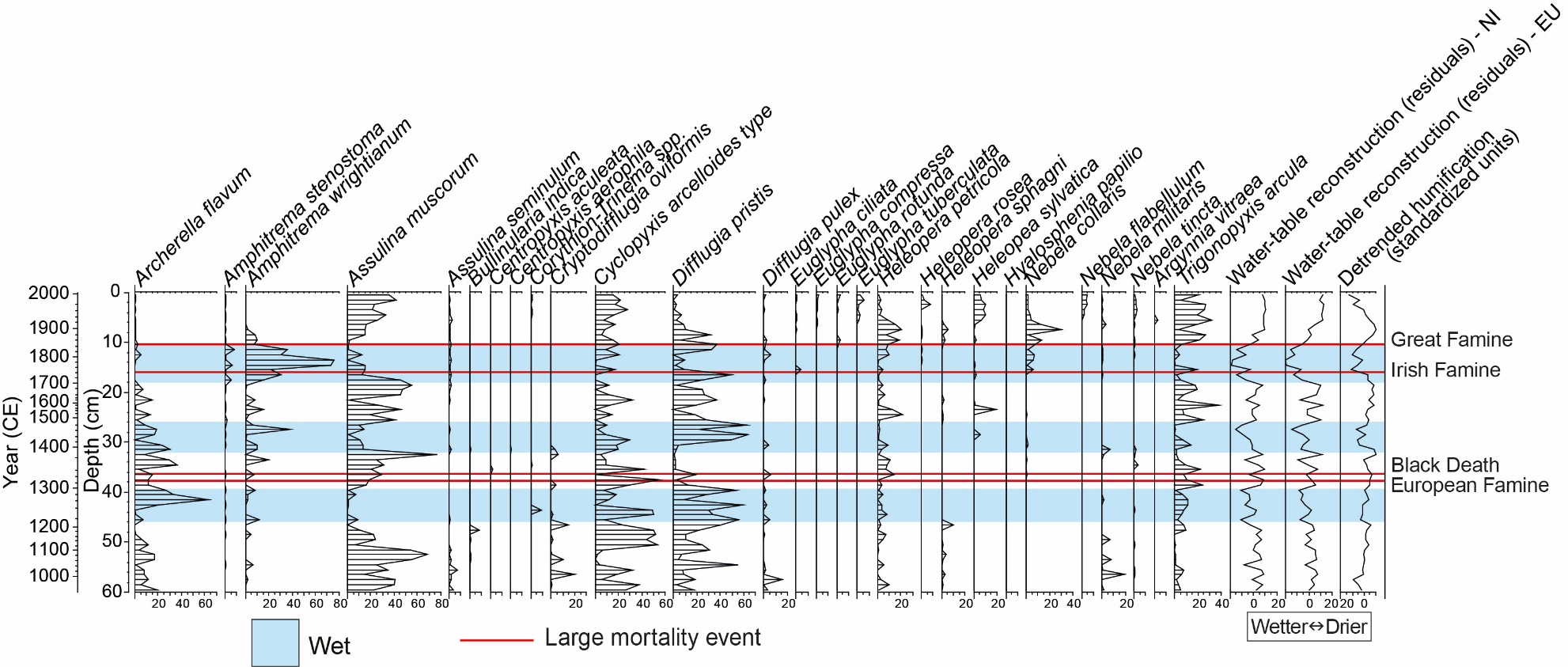


**S4 Fig. Testate amoeba percentage diagram**, showing detrended water-table reconstructions derived from the Northern Ireland (NI) and European (EU) training sets. Phases of wetter bog surface, as determined from the testate amoeba-derived water-table reconstructions and detrended humification (presented as light transmission standardized units) are highlighted (blue bands), as is the timing of major demographic crises (red lines).


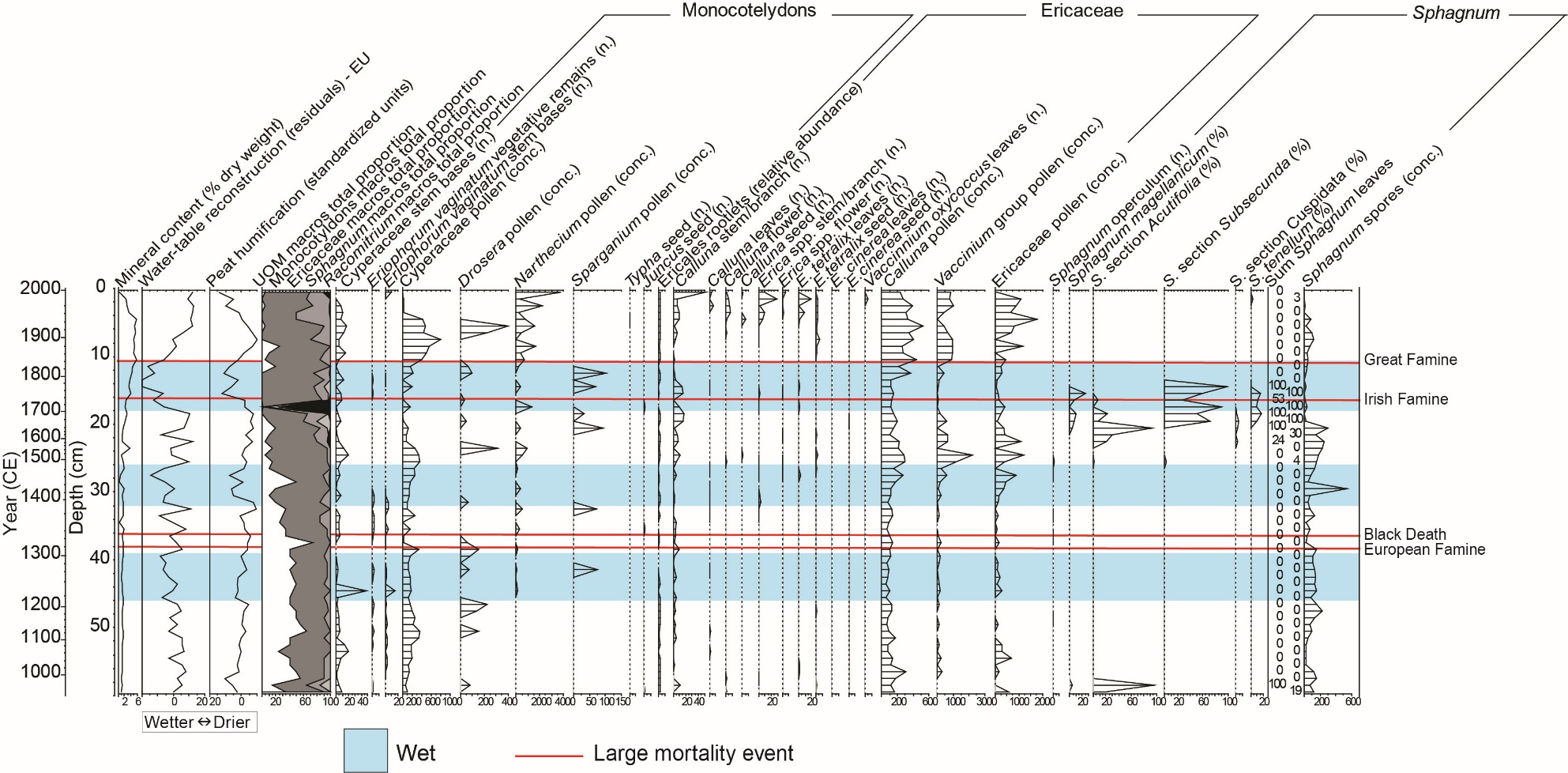


**S5 Fig. Local vegetation indicators at Slieveanorra**, including plant macrofossils and pollen/spore concentrations (grains per cm^3^). The main peat components were recorded as proportions (expressed as percentages), seeds and other identifiable plant parts were counted, and Ericales rootlets were estimated on an abundance scale ranging from 0 (absent) to 5 (dominant). Where *Sphagnum* leaves were present, a maximum of 100 leaves were identified and have been calculated as percentages of the total *Sphagnum* leaf count. Phases of wetter bog surface, as determined from the testate amoeba-derived water-table reconstructions and humification data (presented as detrended light transmission data) are highlighted (blue bands), as is the timing of major demographic crises (red lines). Mineral content of the peat is also shown.

**
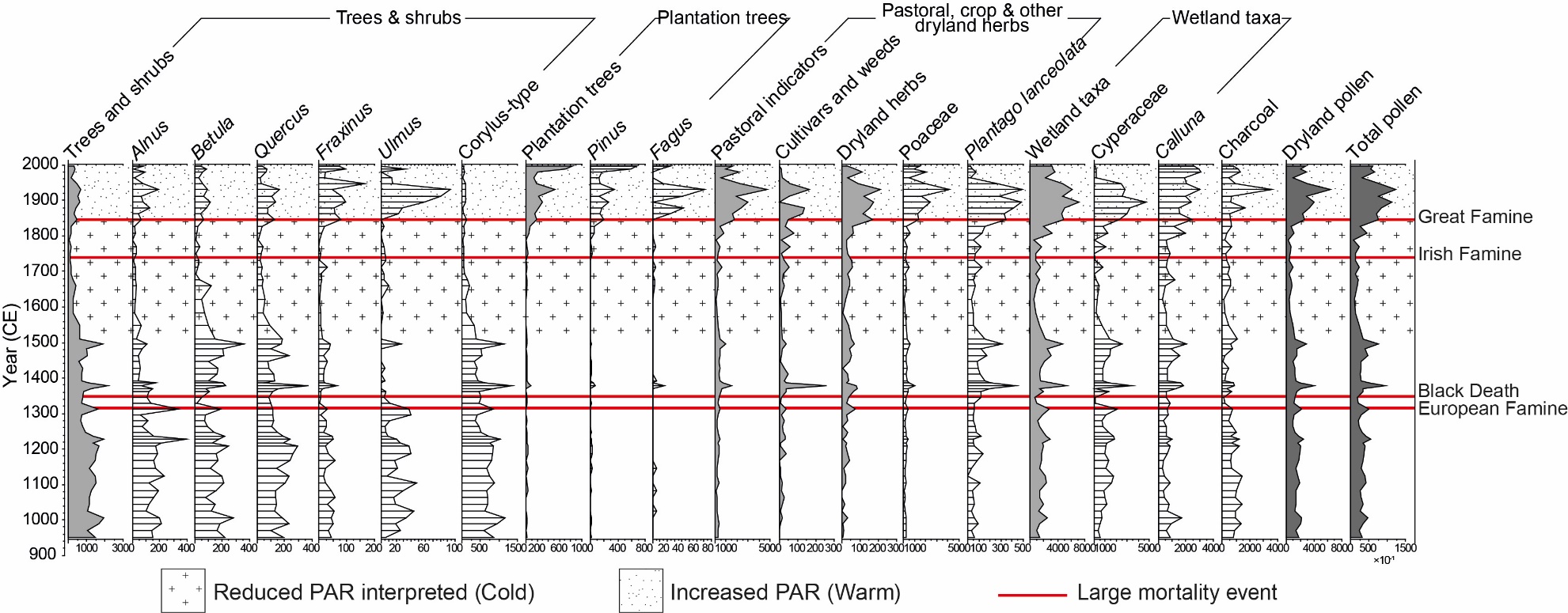
**

**S6 Fig.** **Pollen Accumulation Rates (PAR) diagram (selected taxa and groups) from Slieveanorra, Co. Antrim, Northern Ireland.** Periods of reduced and elevated PAR are highlighted by stippled zones.


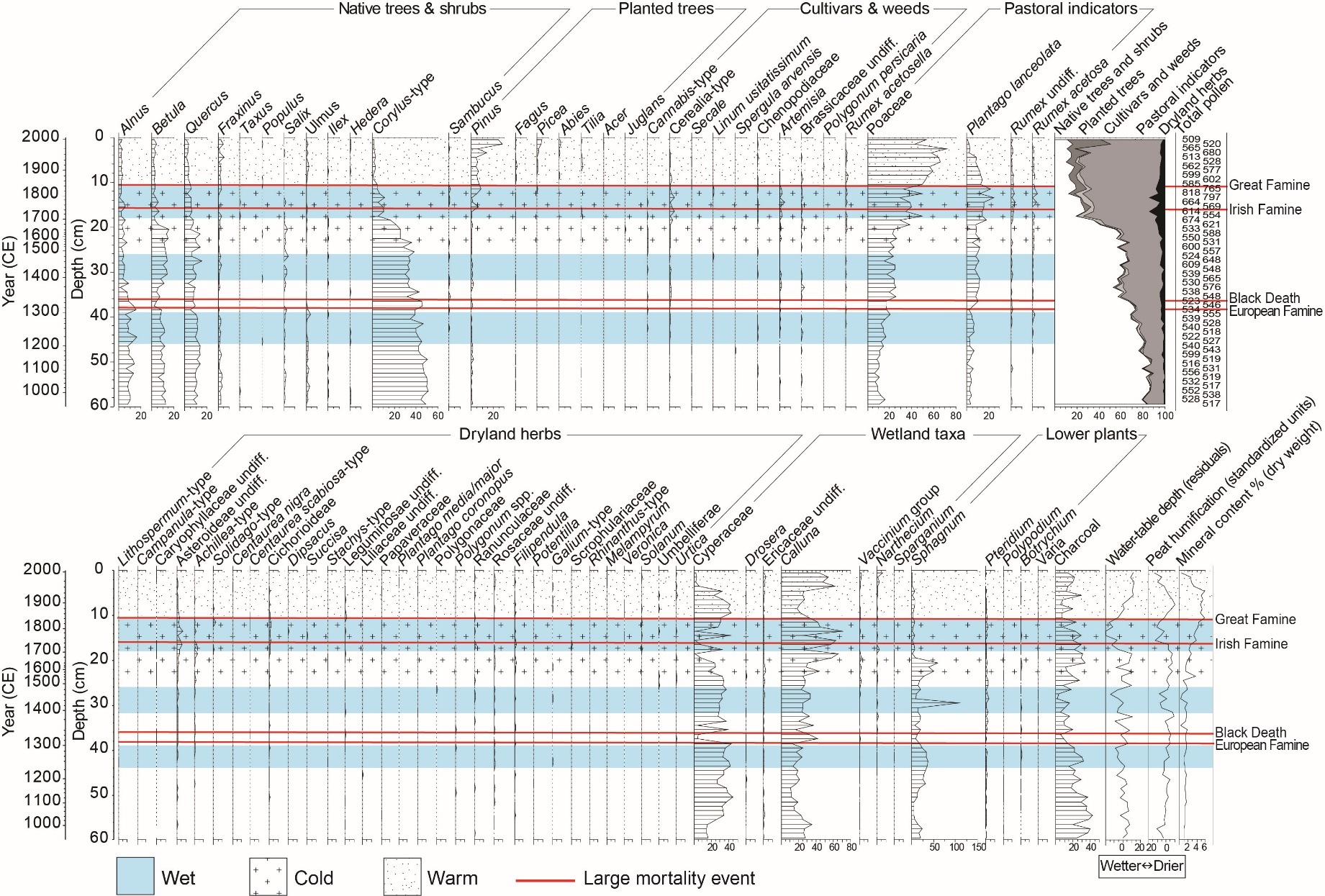


**S7 Fig. Percentage pollen diagram from Slieveanorra, Co. Antrim, Northern Ireland**. Percentages are based on total dryland pollen (excluding bog taxa and spores). Phases of wetter bog surface, as determined from the testate amoeba-derived water-table reconstructions (residuals) and detrended humification data (presented as detrended light transmission data in standardized units) are highlighted (blue bands), while warm and cold periods inferred from pollen accumulation rates are illustrated by stippled zones. The timing of major demographic crises is indicated by red lines. Mineral content of the peat (% dry weight) is also shown.

**References**

1. Swindles GT, Blundell A, Roe HM, Hall VA. A 4500-year proxy climate record from peatlands in the North of Ireland: the identification of widespread summer 'drought phases'? Quat Sci Rev. 2010:29: 1577–1589.
2. Plunkett G, Pilcher JR. Defining the potential source region of volcanic ash in northwest Europe during the Mid-to Late Holocene. Earth-Sci Rev 2018: 179:20–37.
3. Holmquist JR, Finkelstein SA, Garneau M, Massa C, Yu Z, MacDonald GM. A comparison of radiocarbon ages derived from bulk peat and selected plant macrofossils in basal peat cores from circum-arctic peatlands. Quat Geochronology 2016:31: 53–61.
4. Blaauw M, van der Plicht J, van Geel B. Radiocarbon dating of bulk peat samples from raised bogs: non-existence of a previously reported reservoir effect? Quat Sci Rev 2004:23: 1537–1542.
5. Bronk Ramsey C. Deposition models for chronological records. Quat Sci Rev 2008:27: 42–60.
6. Bronk Ramsey C. Bayesian analysis of radiocarbon dates. Radiocarbon 2009*:*51: 337–360.
7. Reimer PJ, Austin WE, Bard E, Bayliss A, Blackwell PG, Ramsey CB, et al. The IntCal20 Northern Hemisphere radiocarbon age calibration curve (0–55 cal kBP). Radiocarbon. 2020;62: 725–757. doi: 10.1017/RDC.2020.41.
